# Supplementary material for: The inevitability of Covid-19 related distress among healthcare workers: Findings from a low caseload country under lockdown
Source: PLoS One. 2021 Apr 1;16(4):e0248741. doi: 10.1371/journal.pone.0248741 (PMC8016221; doi:10.1371/journal.pone.0248741)
Supplement: Attachment — Submitted filename: Response to Reviewers.docx [file pone.0248741.s001.docx]

We would like to thank both reviewers for their time and effort, and for their constructive feedback. We have addressed each point below.

**Reviewer #1:**

**The present study aimed at investigating psychological distress and associated factors in a sample of 1006 Jordanian healthcare workers during a stringent lockdown due to COVID-19 pandemic. Results showed 32% of subjects reporting high distress levels and 20% of subjects suffering very severe distress. Further, around a third and a quarter also reported moderate to severe anxiety and depressive symptoms, respectively, besides another third reporting sleep problems. Authors also reported demographic and other personal characteristics associated to higher distress levels. Particularly, younger age, being female, being pharmacist, working in a cancer hospital, higher workload, suffering burnout or sleep problems, feeling frightened by the virus spreading uncontrollably and by the separation from family and friends were the strongest predictors of higher distress levels. The topic is timely and well structured. However, some considerations that would help to increase the quality of the work could be taken into account.**

**1) The paper definitely needs the revision of a qualified English native speaker.**

We have gone over the manuscript again to ensure it meets PLos One requirements that the language is clear, correct, and unambiguous. We also went through our text to make sure that there are no typographical or grammatical errors, and made general changes to the flow of text as well as the wording of certain sections. If the reviewer has any further concerns, we can address these if specific issues are pointed out.

**2) Authors should provide details on the validity and reliability of each instrument used in the present study. Authors stated that they used only few items of PROMIS questionnaires to assess sleep related impairment and fatigue. The lack of a specific and complete questionnaire to assess these symptoms could represent a limitation when interpreting the present results and should be declared. Further, it could be useful to report which items of these questionnaires were used to the present assessment, describing them in the Methods section.**

We have restructured the Methods section and have added some of this information to it as requested by the reviewer. We could not include exact descriptions of each item in the Methods section (to avoid an overly long section), but we now also include a copy of the survey (the original Arabic survey administered, and an English translation), which should provide readers with details about the specific items used.

We also now discuss our selection of items and the limitations of our approach in the Limitations paragraph in the Discussion.

**3) Moreover, burnout was evaluated by a single-item measure, referring to Dolan et al. study (2015). Firstly, the item and its score should be described in Methods. Secondly, the use of a single-item measure could represent another possible limitation in the quality of burnout symptoms assessment, as reported also by Dolan et al. (2015) in their study, and this should be declared in limitations.**

We have added details to our Methods section, and have updated our Limitations section to include this point.

**4) The paper is interesting because reported data on healthcare workers in a country with low caseloads, confirming the potentially traumatic impact of COVID-19 pandemic on mental health of such population. These data seem to be in line with a recent study assessing burnout, anxiety and depressive symptoms and their association in healthcare workers facing the first phase of COVID-19 pandemic in an Italian region in lockdown with low caseloads (see doi: 10.3390/ijerph17176180). These data could suggest that facing a new and unknown threat was the most stressful factor itself, rather than the number of caseloads. This could be commented when discussing results.**

We agree with the reviewer’s comments, and our original intention was to express this (that facing a new and unknown threat was the most stressful factor itself, rather than the number of caseloads). We have reworded the first part of our Discussion to reiterate the point as suggested above.

We also thank the reviewer for pointing out the study. As we mention in another comment to reviewer 2, at the time that our manuscript was submitted to Plos One (several months ago), the number of studies available on this subject was much smaller than the number available today. We have updated our Background and Discussion to reflect more recent literature.

**5) Results showed reporting effective institutional safety measures in workplace as well as feeling satisfied at work were significantly associated to lower distress levels. Authors could better discuss this point, for example considering a recent systematic review on healthcare workers facing Coronavirus outbreaks (including COVID-19 pandemic studies) reporting perceived safety of the working environment and a good work organization, as factors which seem to protect healthcare workers to the development of work-related posttraumatic stress, as well as a clear communication of directives and supervisors’ and colleagues’ support (see doi: 10.1016/j.psychres.2020.113312). Similarly to the present results, the same study reported as the fear of infecting others, as well as social isolation and family separation where related to higher distress symptoms.**

We thank the reviewer for these comments and have updated our Discussion. In addition, we added three more items to our Descriptive results (Table 1), which we had not originally included to avoid a lengthy table, but we think would be useful for readers to view, particularly in light of what the reviewer mentions about work environment and working relations with colleagues. The added items were “agree that co-workers could be relied on to do their jobs well”, “agree that peers could openly talk about what was and wasn't working” and “agree that sufficient training was provided for use of personal protective equipment”. It is important to note that the variable “agree that peers could openly talk about what was and wasn't working” is now included in the multivariable analysis (the other variables did not contribute to the model significantly).

**Reviewer #2:**

**The main aim of this paper was to evaluated distress levels in healthcare workers during the covid-19 pandemic in Jordan. Moreover, the specific aim was to investigate the factors associated with psychological distress in this population in order to carry out psychological interventions to improve medical professionals’ resilience. Although the topic is interesting, there are some unclear points that must be clarified. The reviewer hopes that the comments below will be helpful to improve the manuscript. The following suggestions are divided into parts.**

**Introduction**

**I would suggest you to improve this section by adding other studies about this topic.**

We agree with the reviewer. At the time that our manuscript was submitted to Plos One (several months ago), the number of studies available on this subject was much smaller than the number available today. We have updated our Background to include the reviews that are now available on the numerous studies relating to the mental health of healthcare practitioners, and we have added more content to our Discussion to reflect this more recent literature.

**Study variables and measures**

**Referring to the following sentence: “Fear statements were originally measured using a 5-point Likert scale (from “not at all” to “a very great extent”) and then dichotomized for analysis, by considering those who responded “to a considerable extent” and “to a very great extent” as fearful regarding the statement.”, it is not clear the choice to exclude subjects who do not experience fear. Please clarify this point.**

We did not exclude subjects who did not experience fear. When we dichotomized our fear items we assumed those who selected “not at all”, “a little” or “somewhat” to not face this source of fear; while those who selected either of the last two points on the Likert scale (reflecting “a lot” of fear) to have harbored this fear.

We have added more details to our Methods section which describes our measurements.

**Statistical analysis: This section is unsatisfactory. A more detailed description of all analysis carried out is recommended. Moreover, the authors, have declared the intention to maintain model parsimony. Despite this, I have many concerns about the solidity of the model. Indeed, a high number of variables has been included in the multivariable ordinal logistic regression.**

Originally, we conducted the relevant diagnostic tests to ensure our model did not suffer from multi-collinearity or quasi-separation; and that it did not violate the assumptions of ordered logistic regression. Nevertheless, we understand the reviewer’s concerns regarding the number of variables. We have now adopted a more parsimonious approach, which we describe in the Statistical Methods section. Specifically, we now only include variables that impact model fit and are significant in the final model. Our revised final model remains largely consistent with our originally submitted one with few differences: age is categorical in the revised model to offer more insight about the effect of age in specific categories, and because categorizing age improved the model fit); the effect of being a pharmacist (relative to a physician) is borderline significant; and after adding the variable “agree that peers could openly talk about what was and wasn't working”, perceived effectiveness of safety measures put in place was no longer significant.

**Finally, the latest analysis (the evaluation of the Access to PPE and other perceptions related to PPE use across different professions) deviate from the main objective and risk burdening the study. I suggest to remove them.**

We have removed this secondary analysis and edited our Methods section accordingly.

**Results**

**The authors point out that the category of nonmedical personnel has been excluded from the analysis. It is therefore not clear why it was included in the final sample. I suggest to take into account the possibility of excluding this category from the final sample.**

This category has now been excluded completely.

**The authors declared that: “Distress levels correlated consistently and significantly with all fear items and with anxiety and depression scores”. The association between distress and anxiety and depression scores seems obvious considering that they represent very overlapping constructs. Have the authors taken this into account?**

We made this statement specifically for the reason the reviewer is suggesting: that they would indeed correlate. Because there are different methods to measure distress, and because some studies measure only anxiety and/or depression (rather than use a distress scale), we wanted to reiterate the overlapping nature of these constructs and ensure that our measurement of distress was indeed correlated with other measures that also reflect mental stress.

**Discussion: I would suggest you to improve this section. The results should be better compared to the literature. There are in fact several other studies about the distress on healthcare workers. Please, see for example Di Tella et al.2020; Castelli et al. 2020.**

We have done so (as we mentioned in an earlier comment, at the time of our submission, the number of studies available on this subject was much smaller). We have updated our manuscript to include more recent literature. The Di Tella et al study in particular is part of a review we now cite.

**I suggest you to move the following sentence “Our results suggest that we need to do more with regards to preparing and protecting our healthcare practitioners in anticipation of the realistic possibility of a future surge in Covid-19, given that our finding suggest that our practitioners are already predisposed and have experienced considerable psychological stress.” to the end of the section and to better argue the clinical implications of the study.**

We have moved this statement and restructured the Discussion in general. We hope that the reviewer finds the changes more conducive to readers.

**Referring to the following sentence “We had originally hypothesized that all pharmacists would experience greater distress…”, it is not clear why the findings are not discussed in relation to the literature (e.g. Basheti et al.)**

We have updated this section and made corrections to the text as we realize the results pertaining to the pharmacists’ section may have been confusing (our discussion was referring to more than one comparison we made but did not present). The section is now more to-the-point and also refers to the study mentioned by the reviewer.
