# Supplementary material for: The inevitability of Covid-19 related distress among healthcare workers: Findings from a low caseload country under lockdown
Source: PLoS One. 2021 Apr 1;16(4):e0248741. doi: 10.1371/journal.pone.0248741 (PMC8016221; doi:10.1371/journal.pone.0248741)
Supplement: Attachment — Submitted filename: Response to Reviewers.docx [file pone.0248741.s002.docx]

We would like to thank the editor and reviewers once again. Our responses are included below.

**I encourage you to pay attention to some grammatical/typographical errors (see for example line 291).**

We reviewed the text again and made minor edits to improve some sentences (we also made a minor edit to Table 2’s title).

**Moreover, I suggest you to eliminate the following sentence “which eventually did take place during the months of September through November of 2020 in Jordan”(lines 468-469).**

This has been removed.

**Finally, since the literature on this subject is getting rich quickly, I suggest you to include this recent review doi.org/10.3389/fpsyg.2020.569935**

We now include this reference. We also reviewed our references to ensure that our bibliography reflects the most up to date information (e.g. if preprints are now publications, they are re-referenced as such now).
